# Repurposing antidiabetic drugs for rheumatoid arthritis: results from a two-sample Mendelian randomization study

**DOI:** 10.1007/s10654-023-01000-9

**Published:** 2023-04-13

**Authors:** Chenxi Qin, Lina-Marcela Diaz-Gallo, Bowen Tang, Yunzhang Wang, Thuy-Dung Nguyen, Arvid Harder, Yi Lu, Leonid Padyukov, Johan Askling, Sara Hägg

**Affiliations:** 1 Department of Medical Epidemiology and Biostatistics, Karolinska Institutet, Stockholm, Sweden; 2 Division of Rheumatology, Department of Medicine Solna, Karolinska Institutet, Karolinksa University Hospital, Stockholm, Sweden; 3 Center for Molecular Medicine, Karolinska Institutet, Karolinska University Hospital, Stockholm, Sweden; 4 Clinical Epidemiology Division, Department of Medicine Solna, Karolinska Institutet, Stockholm, Sweden; 5 Rheumatology, Theme Inflammation and Infection, Karolinska University Hospital, Stockholm, Sweden

**Keywords:** **Rheumatoid arthritis**, **Antidiabetic drugs**, **Mendelian randomization**, **Drug repurposing**

## Abstract

Despite increasing therapeutic options to treat rheumatoid arthritis (RA), many patients fail to reach treatment targets. The use of antidiabetic drugs like thiazolidinediones has been associated with lower RA risk. We aimed to explore the repurposing potential of antidiabetic drugs in RA prevention by assessing associations between genetic variation in antidiabetic drug target genes and RA using Mendelian randomization (MR). A two-sample MR design was used to estimate the association between the antidiabetic drug and RA risk using summary statistics from genome-wide association studies (GWAS). We selected independent genetic variants from the gene(s) that encode the target protein(s) of the investigated antidiabetic drug as instruments. We extracted the associations of instruments with blood glucose concentration and RA from the UK Biobank and a GWAS meta-analysis of clinically diagnosed RA, respectively. The effect of genetic variation in the drug target(s) on RA risk was estimated by the Wald ratio test or inverse-variance weighted method. Insulin and its analogues, thiazolidinediones, and sulfonylureas had valid genetic instruments (n = 1, 1, and 2, respectively). Genetic variation in thiazolidinedione target (gene: *PPARG*) was inversely associated with RA risk (odds ratio [OR] 0.38 per 0.1mmol/L glucose lowering, 95% confidence interval [CI] 0.20–0.73). Corresponding ORs (95%CIs) were 0.83 (0.44–1.55) for genetic variation in the targets of insulin and its analogues (gene: *INSR*), and 1.12 (0.83, 1.49) 1.25 (0.78-2.00) for genetic variation in the sulfonylurea targets (gene: *ABCC8* and *KCNJ11*). In conclusion, genetic variation in the thiazolidinedione target is associated with a lower RA risk. The underlying mechanisms warrant further exploration.

**Supplementary Information:**

The online version contains supplementary material available at 10.1007/s10654-023-01000-9.

## Introduction

Rheumatoid arthritis (RA) is a chronic inflammatory joint disease affecting around 0.5 to 1.0% of the adult population [1]. Therapeutic drugs against RA include synthetic and biological disease-modifying anti-rheumatic drugs (DMARDs), and glucocorticoids [2]. Despite available treatment options, a significant proportion of patients with RA fail to reach low disease activity or remission [2]. Because of lower accessibility to biological DMARDs in low-income countries, the true failure rate may be higher [3].

There are unmet preventive and therapeutic needs for RA. *De novo* drug discovery can be time- and resource-consuming. Drug repurposing is an alternative, which aims to apply licensed drugs, with well-studied safety and pharmacokinetic profiles, to new indications [4]. Established RA has been associated with impaired glucose metabolism, especially insulin resistance [5]. Major drugs to improve glucose metabolism include metformin as the first-line treatment, and glucagon-like peptide-1 (GLP-1) receptor agonists, sodium-glucose cotransporter-2 (SGLT2) inhibitors, dipeptidyl peptidase 4 (DPP-4) inhibitors, insulin and its analogues, thiazolidinediones and sulfonylureas as second-line options [6]. Metformin use was reported to be associated with a decreased risk of developing RA [7]. Likewise, the use of thiazolidinediones, a drug class activating peroxisome proliferator-activated receptor-γ (PPARγ) to reduce glucose concentration and increase insulin sensitivity, was associated with a reduced RA risk [8]. Glucagon-like peptide-1 (GLP-1) is an incretin hormone inducing insulin secretion and suppressing glucagon production [9]. Dipeptidyl peptidase 4 (DPP-4) inhibitors can prevent GLP-1 from degradation and GLP-1 receptor agonists can mimic the physiological function of GLP-1 [9]. A meta-analysis of four retrospective cohorts of patients with type 2 diabetes mellitus (T2DM) found that DPP4 inhibitor users had a 28% lower risk of incident RA compared with those who did not receive DPP4 inhibitors [10]. Likewise, *in vitro* studies found anti-inflammatory effects of GLP-1 receptor agonists in fibroblast-like synoviocytes (FLS) from patients with RA [11, 12]. Evidence is scarce concerning the impact of using SGLT2 inhibitors, insulin and sulfonylureas on RA risk.

**Fig. 1:**
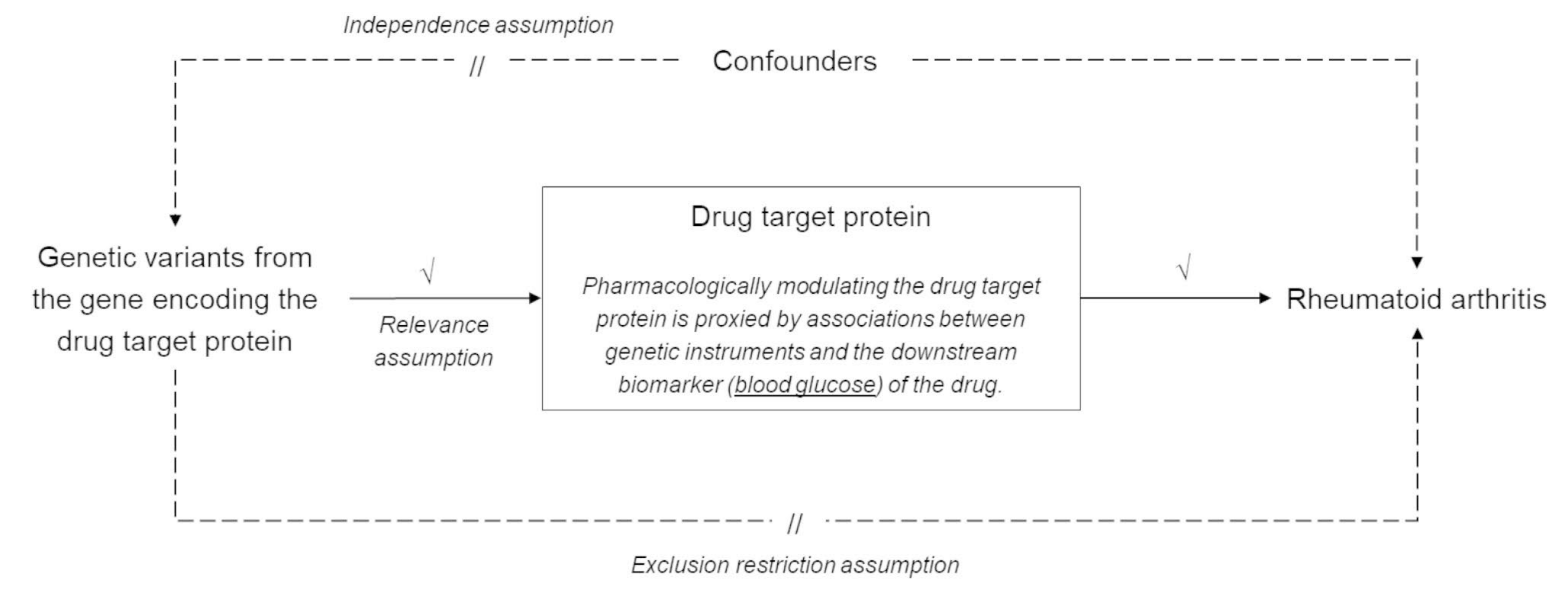
**Overview of the Mendelian randomization study design**. The Mendelian randomization (MR) design uses alleles randomized at germ cell formation and conception as instruments to estimate unconfounded associations between an exposure and an outcome, and can be a viable method to gauge the potential of drug repurposing. This study selects genetic variants from the gene(s) encoding the target protein(s) of the antidiabetic drug as instruments. The main downstream effect of antidiabetic drugs is decreased glucose concentration. Hence, this study leverages associations of the selected genetic instruments with blood glucose concentration to proxy the pharmacological modulation of the drug target protein of interest (relevance assumption). Genetic variants are expected to not be associated with confounders (independence assumption) and affect RA through other pathways (exclusion restriction assumption). The two samples used in the analyses were summary data from genome-wide association studies on blood glucose (UK Biobank) and clinically diagnosed RA.

Because of their observational designs, these studies have demonstrated associations but not necessarily causality, for instance, reverse causation may have been at play. Mendelian randomization (MR) can be a viable approach to gauge the repurposing potential of a drug [13]. Briefly, MR uses genetic variants, usually single nucleotide polymorphisms (SNPs), as instruments of an exposure to provide the ideally unconfounded effect of the exposure on the outcome [14]. Genetic variants from the gene locus that encodes the drug target protein are likely to influence the expression or function of the protein. Leveraging these variants as genetic instruments can mimic how the drug modulates its target protein, allowing us to estimate the effect of genetic variation in the drug target on a new indication like a randomized controlled trial (Fig. 1) [13, 15]. This study aimed to evaluate the repurposing potential of antidiabetic agents to prevent RA in an MR framework, using genetic variants in the encoding gene loci of the targets of different antidiabetic drugs.

## Methods

### Study overview

We utilized a two-sample MR design to provide evidence for the potential usefulness of antidiabetic drug repurposing on RA prevention by deriving summary statistics of instrument-exposure and instrument-outcome associations from large-scale genome-wide association studies (GWAS) in separate populations (Fig. 1). We first identified the gene(s) that encode the target protein(s) of each antidiabetic drug class from Drugbank and ChEMBL databases, and chose independent variants from the gene locus as instruments to proxy the pharmacological modulation of the drug target protein. We extracted associations between genetic instruments and blood glucose concentration from the glucose GWAS in the UK Biobank (UKB)[16], and associations between genetic instruments and RA risk from the so far largest GWAS of clinically diagnosed RA [17]. Then we combined two datasets to estimate the effects of genetic variation in the antidiabetic drug targets on the risk of RA using MR analysis. Details about all the GWAS used in our study are listed in Table 1.

**Table 1:** Details of the genome-wide association studies used in the present study

Trait	Inclusion criteria	Sample size	Exclusion criteria	Measurement/ diagnostic criteria	Covariates	Reference
**Exposure**						
Blood glucose	All participants from UK Biobank	309,895	Individuals with diagnosed or self-reported type-1 or type-2 diabetes, on diabetes treatment	Measured by hexokinase method	Age, sex, array and 10 PCs	*(NA)*
Fasting insulin*	Participants from 77 studies	105,056	Individuals with diagnosed type 1 or type 2 diabetes, with fasting glucose ≥ 7mmol/L, on diabetes treatment, with pregnancy, with outliers ± 3 s.d.; non-fasting individuals	Measured by various enzymatic methods	Age, study site, PCs	[35]
**Outcome**						
RA	Participants from 25 RA studies	22,350 cases/ 74,823 controls	*(NA)*	The 1987 American College of Rheumatology criteria or the 2010 European League Against Rheumatism criteria, or diagnosed RA by a rheumatologist	Age, sex and PCs	[17]
Seropositive RA	Participants from 25 RA studies	17,221 cases/ 74,823 controls	*(NA)*	Diagnosis criteria are the same as above. Seropositivity is defined as the presence of rheumatoid factor or anti-citrullinated peptide antibodies.	Age, sex and PCs	[17]
**Positive control outcomes**						
T2DM	Participants from 18 studies	26,676 cases/ 132,532 controls	*(NA)*	Self-reported diabetes diagnosed by a physician, self-reported antidiabetic drug use, fasting-glucose ≥ 7.0mmol/L, non-fasting glucose ≥ 11.1 mmol/L, HbA1c ≥ 6.5%, a diagnosis of diabetes in the hospital discharge register	Age, sex and PCs	[33]
HOMA-IR	Participants from 20 studies	36,466	Individuals with diagnosed type-1 or type-2 diabetes, with fasting glucose ≥ 7.0mmol/L, on diabetes treatment, with pregnancy, with outliers ± 3 s.d.; non-fasting individuals	HOMA-IR=(fasting insulin [mU/L] × fasting glucose [nmol/L]) × 22.5	Age, sex, study site, geographic covariates and age squared	[34]
Fasting proinsulin	Participants from 16 studies	45,861	Individuals with self-reported or diagnosed diabetes, antidiabetic drug use, fasting-glucose ≥ 7.0mmol/L, 2-hour glucose ≥ 11.1 mmol/L, HbA1c ≥ 6.5%	*(NA)*	Age, sex, PCs and other study-specific covariates	[36]
BMI*	Participants from 125 studies	322,154	Individuals with missing information (including weight, height, BMI, age or sex), with age < 18 years	Self-reported or measured height and weight. BMI = weight (kg) / height (m)^2^	Age, age squared and study-specific covariates such as PCs	[29]
Hip circumference*	Participants from 57 studies	213,038	Individuals with missing information (including hip circumference, weight, height, BMI, age or sex), with age < 18 years	Self-reported or measured metrics	Age, age squared, study-specific covariates	[30]
Waist circumference*	Participants from 57 studies	232,101	Individuals with missing information (including hip circumference, weight, height, BMI, age or sex), with age < 18 years	Self-reported or measured metrics	Age, age squared, study-specific covariates	[30]

PC, principal components; RA, rheumatoid arthritis; T2DM, type 2 diabetes mellitus; HOMA-IR, insulin resistance measured by homeostatic model assessment; BMI, body mass index; NA, not applicable.

All samples are from the European ancestry.

* Genetic associations were estimated in each sex and then combined using meta-analyses.

### GWAS of blood glucose (instrument-exposure associations)

We performed a GWAS analysis of blood glucose concentration in the UKB. Briefly, the UKB recruited about half a million participants aged 40 to 69 years across the UK between 2006 and 2010 [18]. UKB collected a random blood sample for glucose concentration. We excluded participants with self-reported diabetes or diabetes treatment at baseline, and those diagnosed with diabetes (International Classification of Diseases [ICD] 10th code: E10-E14; ICD9 code: 250) before the enrollment, as identified through the linkage to multiple databases (Supplementary Fig. 1). Genotype data after imputation were available in the UKB [16]. We excluded individuals having a high proportion of heterozygous genotypes (the indication of low sample quality), sex chromosome aneuploidy, or > 5% missing rate of variants. To correct for relatedness between individuals, one relative was randomly retained within pairs with a kinship threshold of 0.0884. We removed variants with a missing rate > 0.1 or a minor allele frequency < 0.01. We restricted the analysis to White British participants without missing information on glucose concentration (N = 309,895) and adjusted for age at baseline, sex, array and ten principal genetic components.

### GWAS of rheumatoid arthritis (instrument-outcome associations)

We obtained information on genetic association data of clinically diagnosed RA from a GWAS meta-analysis of 22,350 cases and 74,823 controls from 25 European RA cohorts (downloaded on 2021/12/07; Table 1) [17]. Genetic associations specifically for seropositive RA were also available in this GWAS that encompassed 17,221 seropositive cases. Seropositive RA case was defined as RA positive for either rheumatoid factor or anti-citrullinated peptide antibodies. Covariates included age, sex and principal components, as appropriate.

### Genetic instruments for anti-diabetic drugs

Major antidiabetic drugs included metformin, GLP-1 receptor agonists, SGLT2 inhibitors, DPP-4 inhibitors, insulin and its analogues, thiazolidinediones and sulfonylureas [6]. Genes encoding the target proteins of these antidiabetic drugs were identified from Drugbank (v5.0) and ChEMBL (v29.0) databases (Table 2) [19, 20]. Metformin was removed because two databases listed different target proteins and its drug mechanisms have not been fully clarified.

**Table 2:** Target genes of antidiabetic drugs from DrugBank and ChEMBL databases

Drug class	Encoding genes of target proteins		Gene location
	DrugBank	ChEMBL	
Metformin	*PRKAB1*	Fifty-eight encoding genes	*(NA)*
	*ETFDH*	*GPD2*	
GLP-1 receptor agonists	*GLP1R*	*GLP1R*	Chr6: 39,016,557 − 39,059,079
SGLT2 inhibitors	*SLC5A2*	*SLC5A2*	Chr16: 31,494,323 − 31,502,181
DDP-4 inhibitors	*DPP4*	*DPP4*	Chr2: 162,848,755 − 162,930,904
Insulin and its analogues	*INSR*	*INSR*	Chr19: 7,112,266-7,294,425
Thiazolidinediones	*PPARG*	*PPARG*	Chr3: 12,328,867 − 12,475,855
Sulfonylureas	*KCNJ11*	*KCNJ11*	Chr11: 17,386,719 − 17,410,878
	*ABCC8*	*ABCC8*	Chr11: 17,414,045 − 17,498,441

GLP-1, glucagon-like peptide-1; SGLT2, sodium-glucose cotransporter-2; DPP4, dipeptidyl peptidase-4; NA, not applicable.

The gene location in the human assembly GRCh37 is obtained from https://grch37.ensembl.org.

We extracted genetic variants from the target gene region and its neighboring 2.5 kb window of each drug class, retained those having associations with the glucose concentration with a false discovery rate less than 0.05, and then applied a linkage disequilibrium (LD) clumping (*r*^*2*^ < 0.001) to select nearly independent variants as genetic instruments (Supplementary Table 1). Palindromic variants were allowed with a minor allele frequency below 0.3. For variants absent in the RA GWAS, we used the European panel from the 1000 Genome Project Phase 3 as the reference panel and chose the variant in high LD (*r*^*2*^ > 0.8) with the drug-target-associated variant as a proxy [21]. To examine whether the genetic instruments would affect RA risk through other pathways other than the drug target of interest, we also searched for traits associated with these instruments in the GWAS Catalog [22].

In addition, we selected genetic variants of biological relevance as instruments for thiazolidinediones and sulfonylureas. A common variant (rs1801282) within the *PPARG* gene is a missense variant and results in the substitution of Ala for Pro at position 12 in the PPARγ2-specific exon B. The rs1801282 variant can regulate binding affinity to PPARγ response element and ability to activate transcription [23]. Its minor allele (G) was associated with a lower T2DM risk [24]. The rs757110 variant within the *ABCC8* gene encodes the subunit of sulfonylurea target protein, ATP-sensitive potassium (K_ATP_) channel. The K_ATP_ channel locates on pancreatic β-cell membranes and its inhibition promotes insulin release [25]. The minor allele (A) of rs757110 could facilitate closing the K_ATP_ channel and was associated with decreased 2-hour glucose concentration and T2DM risk [25, 26]. Genetic instruments of biological relevance were independently leveraged in MR analysis.

### Positive control analysis

A positive control MR analysis serves to justify the genetic instruments of the drug by demonstrating the expected effect on the outcome which has an established causal relationship with the drug of interest [27]. The intended indication for antidiabetic drugs is T2DM. Besides their glucose-lowering properties, GLP-1 receptor agonists and SGLT2 inhibitors have weight loss effects and DDP4 inhibitors are neutral to weight change, whereas insulin and its analogues, thiazolidinediones and sulfonylureas could confer weight gain [28]. We leveraged body mass index (BMI), hip circumference and waist circumference and waist-to-hip ratio as additional positive control outcomes [29, 30]. Thiazolidinediones can ameliorate insulin sensitivity, so we used insulin resistance (IR) as a positive control outcome for thiazolidinediones [31]. This positive control analysis excluded the functional variant (rs1801282) as the instrument for thiazolidinediones because its minor allele had a high allele frequency (G = 0.903) in the IR GWAS. Meanwhile, GLP-1 receptor agonists and sulfonylureas can stimulate insulin secretion, so we used fasting proinsulin concentration to reflect the insulin secretion ability of pancreatic β-cells [32]. The primary MR analysis was only performed for antidiabetic drugs that demonstrated expected associations with these positive control outcomes. Details about genetic association data for these positive control outcomes, e.g., sample size, inclusion/exclusion criteria, covariates, are listed in Table 1 [29, 30, 33–36].

### Sensitivity analyses

We conducted a sensitivity analysis for thiazolidinediones. Fasting insulin concentration significantly decreases in obese or type 2 diabetes patients receiving thiazolidinediones in clinical studies [37, 38]. Hence we additionally used the fasting insulin concentration as a downstream biomarker of thiazolidinediones. We obtained effect sizes of associations between the selected genetic instruments for thiazolidinedione and fasting insulin concentration from the largest GWAS meta-analysis of fasting insulin, which involved 105,056 white European individuals from 38 studies [35]. The GWAS analysis was performed in each sex and then pooled together using meta-analysis. Covariates included age, study site and principal components, as appropriate. Then we repeated MR analysis to estimate the effect of genetic variation in the thiazolidinedione target, which was predicted by insulin decrement, on RA risk. The minor allele frequency of the functional variant (rs1801282) was high (MAF_G_=0.874) in the fasting insulin GWAS so it was excluded from this sensitivity analysis. Additionally, a relaxed clumping threshold (*r*^*2*^ < 0.1) was applied to include more genetic instruments for the primary MR analyses.

### Statistical methods

The effect size of the genetic association between each genetic instrument and the blood glucose concentration was estimated by the linear regression using PLINK v1.9 [39]. The mean *F* statistic was calculated to test the genetic instrument strength for each drug class [40]. An *F* statistic above ten typically indicates a strong instrument [40]. If the effects of the genetic instrument on the exposure and the outcome did not correspond to the same allele on the same DNA strand, we aligned the allele in the outcome dataset to that in the exposure dataset and flipped its genetic effect size accordingly. If a single genetic instrument was obtained, the causal effect was estimated by the ratio of genetic associations with RA and blood glucose concentration (Wald ratio test). If multiple genetic instruments were selected, inverse-variance weighted (IVW) method was used by combining ratio estimates for all instruments in a fixed effect meta-analysis [41]. Furthermore, MR Egger regression, weighted median and weighted mode methods were used to relax the IVW assumption that the average pleiotropic effect was zero [27]. We set the significance level at 0.008 (0.05/6 drug classes) after Bonferroni correction. The MR estimate was scaled to the odds ratio (OR) of RA per 0.1mmol/L lower blood glucose concentration. MR analyses were completed using the R package ‘TwoSampleMR’ [42].

For drugs that emerged as causally related to RA risk in the MR analysis, we checked whether blood glucose concentration and RA colocalized within the gene region of the drug target protein in question using a Bayesian framework [43]. We set the prior probability of the association between each variant with either trait to be 1 × 10^− 4^ and the prior probability of a shared causal variant between two traits to be 1 × 10^− 5^ (R package ‘coloc’) [43].

### Standard protocol approvals, registrations, and patient consents

This research has been conducted using the UK Biobank Resource under Application Number ‘22224’. The UK Biobank has approval from the North West Multi-center Research Ethics Committee (MREC) in the UK and the aim of this study has the approval from the Regional Ethics Review Board in Stockholm. All cohort data included in the GWAS used in the present study have individual approvals from relevant ethical review boards.

## Results

### Genetic instruments for antidiabetic drugs

The blood glucose GWAS was performed in 309,895 UKB participants (Manhattan plot and Q-Q plot in Supplementary Fig. 2). We identified one variant for each of GLP-1 receptor agonists, insulin and thiazolidinediones, and two variants for sulfonylureas, respectively, as instruments for the pharmacological modulation of their corresponding drug target proteins (Supplementary Table 1). No valid genetic instruments were found for SGLT2 and DDP4 inhibitors. Mean *F* statistics of instruments were 19.3 for GLP-1 receptor agonists, 17.4 for insulin, 14.2 for thiazolidinediones and 31.4 for sulfonylureas, respectively. Corresponding *F* statistics for the two instruments of biological relevance were 11.0 for thiazolidinediones and 48.5 for sulfonylureas, respectively. These indicated that strong genetic instruments were chosen for each drug type.

We searched for traits associated with these instruments in the GWAS Catalog to examine if the exclusion restriction assumption was violated. The rs35240997 variant (the instrument for thiazolidinediones) was linked to red blood cell count and high-density lipoprotein (HDL) cholesterol and the functional variant rs1801282 additionally showed associations with triglycerides, systolic blood pressure, sex hormone-binding globulin and serum albumin. The rs5219 variant (the instrument for sulfonylureas) was additionally associated with cortical surface area and blood pressure (Supplementary Table 2).

### Positive control analysis

Genetic variations in the targets of insulin and its analogues, thiazolidinedione and sulfonylurea were associated with a risk reduction in T2DM, with ORs (95%CI) per 0.1mmol/L glucose lowering being 0.40 (0.25–0.65), 0.30 (0.18–0.50) and 0.48 (0.37–0.63), respectively (Supplementary Fig. 3, panel A). Corresponding associations for thiazolidinediones and sulfonylureas were similar when using their functional variants as instruments. In contrast, genetic variation in the GLP-1 receptor agonist target did not predict a deceased T2DM risk (OR 1.25, 95%CI 0.81–1.92). Overall, genetic variation in target(s) of thiazolidinediones, of sulfonylureas, and of insulin and its analogues demonstrated associations with higher risk of obesity-related phenotypes (Supplementary Fig. 3, panel B-D). However, the positive associations between genetic variation in the target of GLP-1 receptor agonists and all obesity-related phenotypes were inconsistent with existing evidence that GLP-1 receptor agonists have a weight loss effect. We particularly treated IR as the positive control outcome for the genetic instrument selected for thiazolidinediones and observed an inverse association between the two (Supplementary Fig. 3, panel E). Genetic variation in the sulfonylureas target was associated with increased insulin secretion (Supplementary Fig. 3, panel F). In summary, positive control analyses justified the selected genetic instruments of insulin and its analogues, thiazolidinediones and sulfonylureas, but not the instrument of GLP-1 receptor agonists.

### Mendelian randomization and sensitivity analysis

**Fig. 2:**
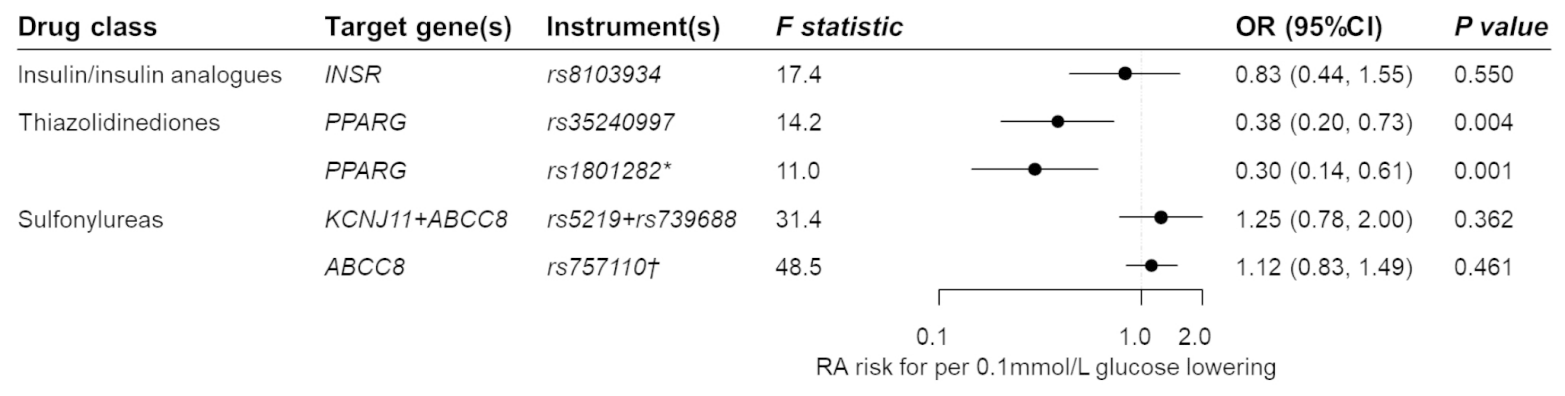
**Estimated effects of genetic variations in antidiabetic drug targets on rheumatoid arthritis**. OR, odds ratio; CI, confidence interval; RA, rheumatoid arthritis. * The rs1801282 variant is a functional variant within the *PPARG* gene region and can regulate binding affinity to PPARγ (encoded by the *PPARG* gene) response element and ability to activate transcription. † The rs757110 variant is a functional variant within the *ABCC8* gene. It can promote insulin release by inhibiting ATP-sensitive potassium channel, of which the subunit is encoded by the *ABCC8* gene. Genetic instruments from the gene region encoding the drug target protein can proxy the antidiabetic drug of interest. Combining variant-glucose and variant-RA associations, the effect of the antidiabetic drug on RA is estimated by the Wald ratio test or inverse-variance weighted method. MR estimates are scaled to RA risk per 0.1 mmol/L glucose lowering

Genetic variation in the thiazolidinedione target (gene: *PPARG*) was significantly associated with a RA risk after multiple testing correction (OR 0.38 per 0.1mmol/L glucose lowering, 95%CI 0.20–0.73, *P* = 0.004; Fig. 2). The association was consistent when using the functional variant rs1801282 as the genetic instrument (OR 0.30, 95%CI 0.14–0.61, *P* = 0.001). By contrast, genetic variation in the targets of insulin and its analogues, and of sulfonylurea, were not associated with RA risk (OR [95%CI]: 0.83 [0.44–1.55] and 1.25 [0.78-2.00], respectively).

**Fig. 3:**
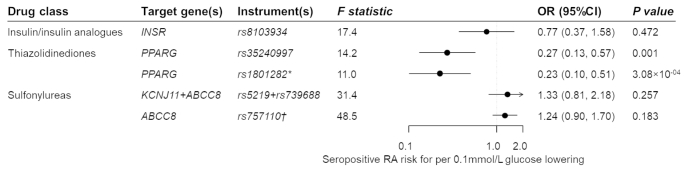
**Estimated effects of genetic variation(s) in antidiabetic drug targets on seropositive rheumatoid arthritis**. OR, odds ratio; CI, confidence interval; RA, rheumatoid arthritis. * The rs1801282 variant is a functional variant within the *PPARG* gene region and can regulate binding affinity to PPARγ (encoded by the *PPARG* gene) response element and ability to activate transcription. † The rs757110 variant is a functional variant within the *ABCC8* gene. It can promote insulin release by inhibiting ATP-sensitive potassium channel, of which the subunit is encoded by the *ABCC8* gene. Genetic instruments from the gene region encoding the drug target protein can proxy the antidiabetic drug of interest. Combining variant-glucose and variant-seropositive RA associations, the effect of the antidiabetic drug on RA risk is estimated by the Wald ratio test or the inverse-variance weighted method. MR estimates are scaled to RA risk per 0.1 mmol/L glucose lowering

When the MR analyses were restricted to seropositive RA, genetic variation in thiazolidinedione target was associated with an OR of 0.27 per 0.1mmol/L glucose decrement for seropositive RA risk (95%CI 0.13–0.57, *P* = 0.001; Fig. 3). Consistent association was observed using the functional genetic instrument (OR 0.23, 95% 0.10–0.51, *P* = 3.08 × 10^− 4^). Corresponding ORs (95%CIs) were 0.77 (0.37–1.58) for insulin and its analogues and 1.33 (0.81–2.18) for sulfonylureas.

We also utilized the fasting insulin concentration as the downstream biomarker of thiazolidinediones and repeated MR analysis using genetic associations of the thiazolidinedione instrument with fasting insulin and RA. Here, genetic variation in the thiazolidinedione target, which was predicted by the decrement of fasting insulin concentration, was associated with lower risks for RA (OR 0.76 per log-transformed unit of lower fasting insulin, 95%CI 0.63–0.91) and seropositive RA 0.69 (95%CI 0.56–0.85). To avoid chance finding due to few instruments, we used a relaxed clumping threshold (*r*^*2*^ < 0.1) to select more genetic instruments and MR analyses showed robust results (Supplementary Table 3).

### Colocalization analysis

The probability of a causal variant associated with glucose concentration, or RA risk, within the *PPARG* gene was 0.7%, and 60.6%, respectively (Supplementary Table 4). The probability of the existence of distinct causal variants between two traits was 1.1% and the probability of a shared causal variant was only 1.9%. The regional association plots of both traits showed similar pattern around the genetic instrument of thiazolidinediones (Supplementary Fig. 4).

## Discussion

This is the first MR study to investigate whether genetic variations in antidiabetic drug targets were associated with RA risk. Leveraging existing large-scale genetic association data on RA risk, our genetic investigation suggests that thiazolidinediones might exert protective effects on RA risk, at least seropositive RA. By contrast, no such association was observed with other antidiabetic drugs.

Our MR findings suggested a causal relationship between genetic variation in the target of thiazolidinediones and RA, but colocalization results did not find a shared causal variant between the glucose concentration and RA within the *PPARG* gene. These seemingly discrepant results may stem from the different methodological theories of two methods: MR selects variants associated with the exposure whereas colocalization is more conservative by requiring significant associations of the causal variant with both traits [44]. The regional association plots of both traits exhibited weak genetic associations of the instrument for thiazolindinediones, which could lead to a weak observed colocalization. Other study designs have reported that exposure to thiazolidinediones was related to a lower RA risk. A population-based case-control study compared the use of thiazolidinediones between T2DM patients with and without an RA diagnosis, and observed an RA risk reduction, although without reaching statistical significance, associated with thiazolidinedione use compared with non-use (OR 0.91, 95%CI 0.81–1.02) [8]. After further excluding participants who took thiazolidinediones 90 days before the diagnosis of RA from the case group, there was a dose-response relationship, with a 25% (95%CI 11-39%) lower RA risk among patients exposing to the highest cumulative defined daily doses of thiazolidinediones [8]. Their finding about lower RA risk in thiazolidinedione users is concordant with our results that genetic variation in the thiazolidinedione target is preventive against RA.

The mechanism behind any preventive effect of thiazolidinediones on RA risk is unclear, but experimental data regarding the therapeutic effects of thiazolidiendiones could provide some insights. PPARγ was remarkably downregulated in FLS from patients with RA compared with FLS from healthy controls [45]. FLS are a dominant cell population in synovium. They can secrete pro-inflammatory cytokines like interleukin-6, interact with macrophage-like synoviocytes and other immune-related cells, and subsequently cause inflamed joints [46]. Murine models have observed that upregulating PPARγ could inhibit the proliferation and migration of FLS [45]. Patients with RA also upregulated PPARγ in peripheral macrophages in comparison with healthy controls, and the extent of PPARγ expression was negatively related to RA disease activity.[47] In the joint pathology of RA, activated macrophages can produce various pro-inflammatory factors and interact with other immune cells [48]. Thiazolidinediones can activate PPARγ and may repress gene transcription by negatively interfering with other transcription-factor pathways to achieve anti-inflammatory effects [31]. Crossover trials also reported that thiazolidinediones could decrease the disease activity in non-diabetic patients who received stable DMARDs treatment for RA [49, 50]. Yet in these studies it was uncertain whether PPARγ dysregulation was driven by the natural pathological course of the RA disease itself, by DMARD treatment, or by other factors [46, 47]. Our results thus highlight the potential for further mechanistically oriented research on the role of PPARγ in RA and other states of chronic inflammation.

To the best of our knowledge, there is no prior research on the impact of insulin, its analogues and sulfonylureas on RA risk. Irrespective, our study did not support a causal effect of insulin and sulfonylureas on RA risk, but on the other hand suggest that whatever mechanism that drives the association between the genetic variation in the thiazolidinedione target and RA risk is not shared with any glucose-lowering drugs, or mediated via other mechanisms shared by the genetic variation in targets of these drugs.

This study has several strengths. Firstly, we applied an MR design to make a causal inference without confounding bias and reverse causation. Secondly, we restricted analyses within the European ancestry to avoid spurious associations due to population stratification. Thirdly, we specifically chose genetic variants from within a narrow window (2.5 kb) of the encoding gene as instruments, which may be related to the gene function or expression. Fourthly, we performed positive control analyses that predicted the effects of antidiabetic drugs on the intended indication and other established outcomes to justify the validity of selected genetic instruments. Finally, the large *F* statistic indicated the small chance of a weak instrument bias.

Yet a few limitations should be acknowledged. Firstly, this study could only predict the drug effect on RA risk via perturbing documented proteins (on-target effects). We could not rule out the possibility of drugs modifying RA risk through other proteins (off-target effects). Secondly, few genetic instruments could lead to a chance finding. However, our study selected genetic instruments of biological relevance for thiazolidinediones and sulfonylureas and validated them using various positive control outcomes. We relaxed the clumping threshold to obtain more genetic instruments and observed robust MR estimates. For insulin and its analogues, GLP-1 receptor agonists, DDP4 inhibitors and SGLT2 inhibitors, MR study could use genetic instruments that are associated with the drug target protein or other downstream biomarkers to estimate the drug effect on RA risk when more relevant data emerge. Thirdly, horizontal pleiotropy could bias MR estimates, but we selected variants from the vicinity of gene locus that are unlikely to have pleiotropic effects through other genes. Regional association plots also showed that variants in high LD with the instrument fell within the *PPARG* gene locus. We found that instruments for thiazolidinediones were related to traits like red blood cell count, HDL cholesterol, triglycerides, blood pressure, sex hormone-binding globulin and serum albumin. But these traits could be vertical pleiotropic effects because thiazolidinediones have been reported to decrease red blood cell count [51], increase HDL concentration and triglycerides [31], lower blood pressure [52], increase sex hormone-binding globulin [53], and reduce albuminuria [54]. Fourthly, the MR estimate reflects the life-long exposure to a drug, while the drug usually exerts their impact in a short window. This means effect sizes in our study may not be comparable to those reported in trials or observational studies. Fifthly, we could not explore whether genetic variation in the thiazolidinedione target has a beneficial effect on seronegative RA because the GWAS of seronegative RA is lacking. Sixthly, we excluded T2DM patients and individuals on antidiabetic agents in the glucose GWAS to avoid the confounding bias from antidiabetic treatments. But this could inflate the ‘healthy volunteer’ bias in the UKB population and therefore lead to null colocalization findings. Finally, analyses within the European ancestry limited the generalizability of our findings to other ethnicities.

## Conclusion

The present study provides MR evidence about thiazolidinediones as a potential drug class for RA prevention. Genetic evidence offers valuable insights into therapeutic development because the genetic link between a drug target and an indication substantially expedites prioritizing drugs and lowers failure rates in clinical trials [55, 56]. Hence, thiazolidinediones and its target protein should be prioritized as a potential therapeutic target to prevent RA. Further studies could incorporate large-scale GWAS of RA progression phenotypes to assess the disease-modifying potential of thiazolidinediones or even the combined effect of DMARDs and thiazolidinediones on RA.

## Electronic supplementary material

Below is the link to the electronic supplementary material.

Supplementary Material 1
