# Supplementary material for: Repurposing antidiabetic drugs for rheumatoid arthritis: results from a two-sample Mendelian randomization study
Source: Eur J Epidemiol. 2023 Apr 13;38(7):809–19. doi: 10.1007/s10654-023-01000-9 (PMC10276071; doi:10.1007/s10654-023-01000-9)
Supplement: Supplementary file 1 — Supplementary Material 1 [file 10654_2023_1000_MOESM1_ESM.docx]

**Supplementary Table 1. Genetic variants as instruments to proxy the perturbation of antidiabetic drug targets**

| **Exposure** | **SNP** | **Chromosome: position** | **Effect allele** | **Other allele** | **Effect allele frequency** | **Beta coefficient** | **Standard error** | ***P* value** | ***F* statistic** |
| --- | --- | --- | --- | --- | --- | --- | --- | --- | --- |
| GLP-1 receptor agonists | rs1076733 | 6: 39045908 | A | G | 0.46 | -0.0070 | 0.0016 | 1.12E-05 | 19.3 |
| Insulin/insulin analogues | rs8103934 | 19: 7189096 | T | C | 0.09 | -0.0115 | 0.0027 | 3.07E-05 | 17.4 |
| Thiazolidinediones | rs35240997 | 3: 12379351 | G | A | 0.21 | -0.0073 | 0.0019 | 1.63E-04 | 14.2 |
|  | rs1801282* | 3: 12393125 | G | C | 0.12 | -0.0080 | 0.0024 | 9.32E-04 | 11.0 |
| Sulfonylureas | rs5219 | 11: 17409572 | T | C | 0.39 | -0.0118 | 0.0016 | 9.34E-13 | 15.7 |
|  | rs739688 | 11: 17427557 | T | C | 0.35 | -0.0056 | 0.0016 | 6.84E-04 |  |
|  | rs757110† | 11: 17418477 | C | A | 0.36 | 0.0115 | 0.0017 | 3.37E-12 | 48.5 |

GLP-1, glucagon-like peptide-1; SNP, single nucleotide polymorphism.

* The rs1801282 variant is a functional variant within the *PPARG* gene region. It can regulate binding affinity to PPARγ (encoded by the *PPARG* gene) response element and ability to activate transcription. This genetic instrument will be independently used in MR analysis to estimate the drug effect on various outcomes.

† The rs757110 variant is a functional variant within the *ABCC8* gene. It can promote insulin release by inhibiting ATP-sensitive potassium channel, of which the subunit is encoded by the *ABCC8* gene. This genetic instrument will be independently used in MR analysis to estimate the drug effect on various outcomes.

**Supplementary Table 2. Traits associated with genetic instruments of antidiabetic drugs**

| **Drug class** | **SNP** | **Associated traits in the GWAS Catalog** |
| --- | --- | --- |
| GLP-1 receptor agonists | rs1076733 | *(NA)* |
| Insulin/insulin analogues | rs8103934 | *(NA)* |
| Thiazolidinediones | rs35240997 | Red blood cell count, hemoglobin concentration, high-density lipoprotein cholesterol |
|  | rs1801282* | Type 2 diabetes mellitus, fasting insulin, body mass index, triglyceride, systolic blood pressure, pulse pressure, calcium, sex hormone-binding globulin, total blood protein, serum albumin |
| Sulfonylureas | rs5219 | Type 2 diabetes mellitus, body mass index, cortical surface area, systolic blood pressure, pulse pressure |
|  | rs739688 | *(NA)* |
|  | rs757110* | Type 2 diabetes mellitus, waist-to-hip ratio, antidiabetic medication use, hemoglobin concentration |

GLP-1, glucagon-like peptide-1; SNP, single nucleotide polymorphism; NA, not applicable.

Traits related to genetic instruments are searched in GWAS Catalog (https://www.ebi.ac.uk/gwas).

**Supplementary Table 3. Estimated effects of genetic variation in antidiabetic drug targets on rheumatoid arthritis using a relaxed clumping threshold**

| **Drug class** | **Gene** | **Outcome** | **No. of SNPs** | **IVW** | **MR Egger** | | | **Weighted median** | **Weighted mode** |
| --- | --- | --- | --- | --- | --- | --- | --- | --- | --- |
|  |  |  |  | **OR (95%CI)** | **OR (95%CI)** | **Intercept** | ***P* for intercept** | **OR (95%CI)** | **OR (95%CI)** |
| Insulin/insulin analogues | *INSR* | RA | 4 | 1.08 (0.75, 1.55) | 0.68 (0.26, 1.77) | -0.028 | 0.416 | 0.97 (0.65, 1.43) | 1.01 (0.69, 1.47) |
|  |  | Seropositive RA | 4 | 1.10 (0.78, 1.55) | 0.76 (0.31, 1.90) | -0.023 | 0.482 | 1.05 (0.67, 1.64) | 1.07 (0.67, 1.71) |
| Thiazolidinediones | *PPARG* | RA | 2 | 0.48 (0.25, 0.93) | *(NA)* | *(NA)* | *(NA)* | *(NA)* | *(NA)* |
|  |  | Seropositive RA | 2 | 0.37 (0.15, 0.88) | *(NA)* | *(NA)* | *(NA)* | *(NA)* | *(NA)* |
| Sulfonylureas | *KCNJ11+ABCC8* | RA | 8 | 1.22 (0.96, 1.56) | 0.87 (0.42, 1.78) | -0.028 | 0.358 | 1.13 (0.89, 1.44) | 1.11 (0.87, 1.43) |
|  |  | Seropositive RA | 8 | 1.36 (1.01, 1.83) | 0.97 (0.39, 2.40) | -0.028 | 0.462 | 1.20 (0.91, 1.59) | 1.20 (0.90, 1.60) |

IVW, inverse-variance weighted; SNP, single nucleotide polymorphism; OR, odds ratio; CI, confidence interval; NA, not applicable.

This sensitivity analysis uses genetic instruments selected based on a relaxed clumping threshold (linkage disequilibrium *r^2^*<0.1). If two genetic instruments are selected, only the IVW method is used to estimate the effect of genetic variation in the drug target gene on rheumatoid arthritis. If more than two genetic instrument are selected, MR Egger, weighted median and weighted mode methods are also used. MR Egger intercept refers to the deviation of the average pleiotropic effect from zero and is tested (*H_0_*: the intercept=0).

**Supplementary Table 4. Colocalization analysis of glucose concentration and rheumatoid arthritis in the *PPARG* gene**

| **Drug class** | **Target gene** | **No. of SNPs** | **PP.H_0_** | **PP.H_1_** | **PP.H_2_** | **PP.H_3_** | **PP.H_4_** |
| --- | --- | --- | --- | --- | --- | --- | --- |
| Thiazolidinediones | *PPARG* | 358 | 35.7% | 0.7% | 60.6% | 1.1% | 1.9% |

SNP, single nucleotide polymorphism;

PP.H_0_, PP.H_1_, PP.H_2_, PP.H_3_ and PP.H_4_ represent the probability of no shared causal variant, a causal variant for glucose concentration, a causal variant for RA, distinct causal variants between two traits and a shared causal variant between two traits in the *PPARG* gene.


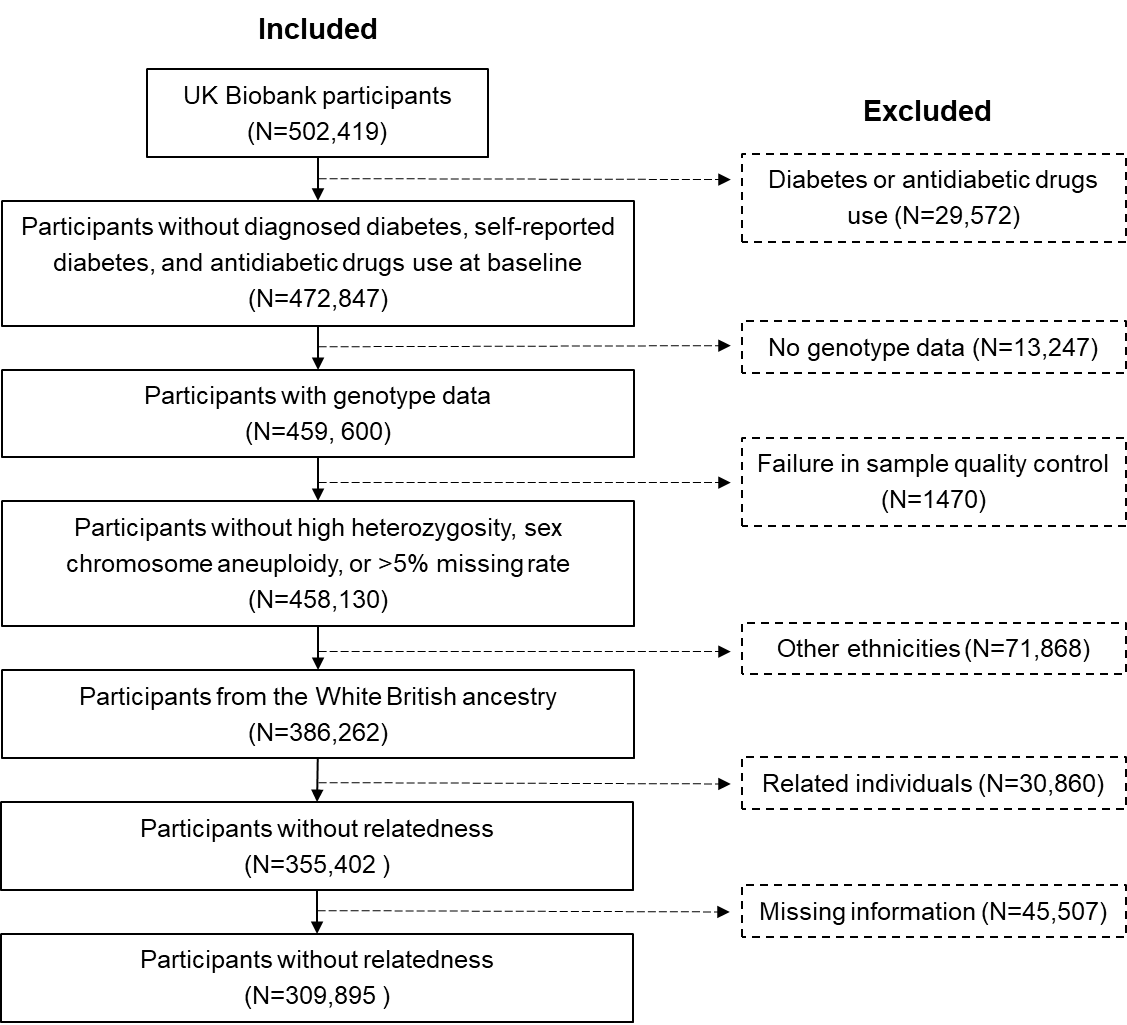


**Supplementary Fig. 1. Flow chart of study population for the genome-wide association study of blood glucose concentration in the UK Biobank**

1. (B)

**
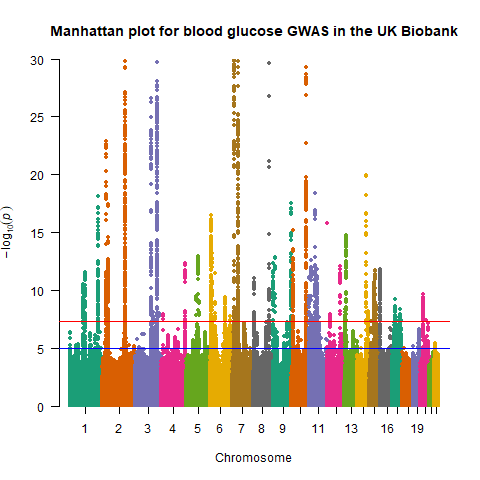

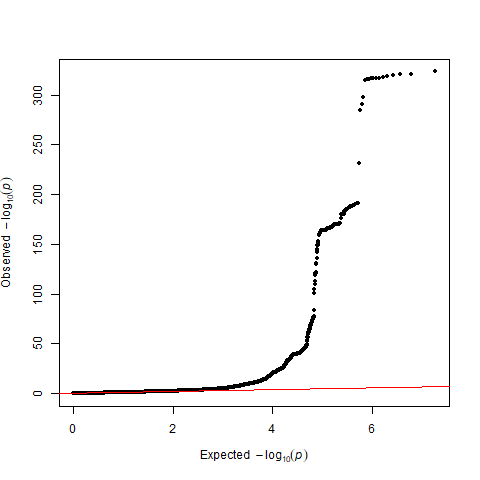
**

**Supplementary Fig. 2. Manhattan plot and Q-Q plot of the genome-wide association study of blood glucose concentration in the UK Biobank (n=309,895)**

(A) Manhattan plot, (B) Q-Q plot.

The Manhattan plot only displays genetic associations on the autosomes. *P* values for the genetic associations are truncated to 1×10^-30^ if they are smaller than 1×10^-30^. The lambda for genomic inflation is 1.164.

**
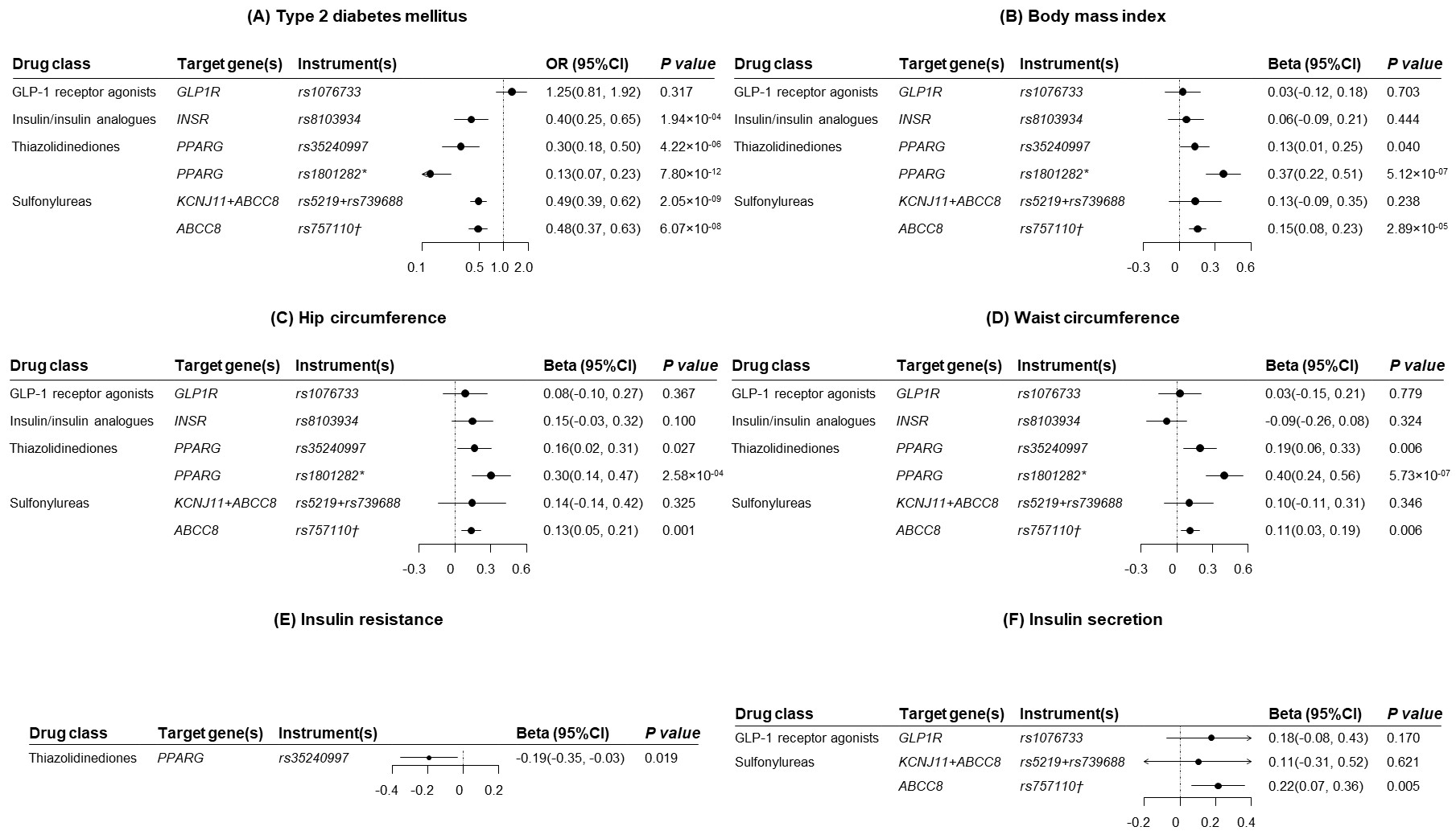
**

**Supplementary Fig. 3. The effects of genetic variations in antidiabetic drug targets on positive control outcomes**

SNP, single nucleotide polymorphism; OR, odds ratio; CI, confidence interval; GLP-1, glucagon-like peptide-1.

* The rs1801282 variant is a functional variant within the *PPARG* gene region and can regulate binding affinity to PPARγ (encoded by the *PPARG* gene) response element and ability to activate transcription.

† The rs757110 variant is a functional variant within the *ABCC8* gene and can promote insulin release by inhibiting ATP-sensitive potassium channel, of which the subunit is encoded by the *ABCC8* gene.

Genetic instruments from the gene region encoding the drug target protein can proxy the antidiabetic drug of interest. Combining SNP-glucose and SNP-outcome associations, the glucose-lowering effect of the antidiabetic drug on the positive control outcome is estimated by the Wald ratio test. MR estimates are scaled to outcome risk per 0.1 mmol/L glucose lowering.

1. (B)


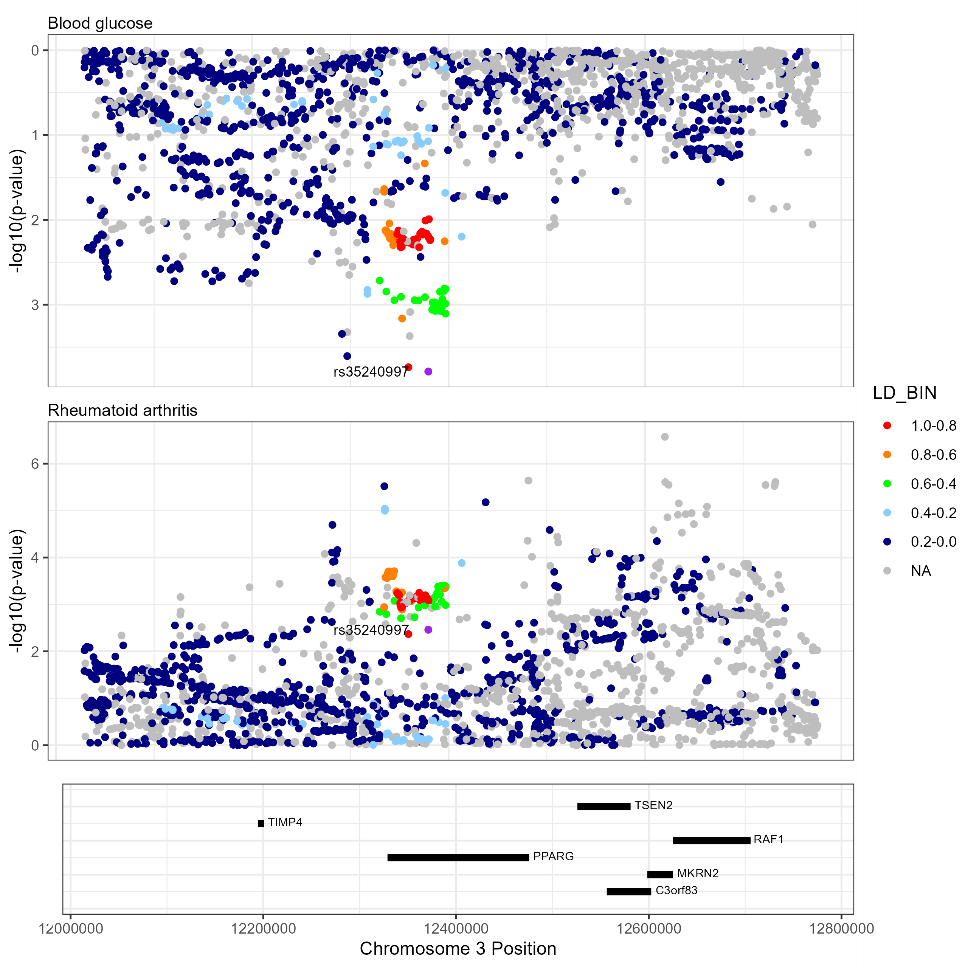

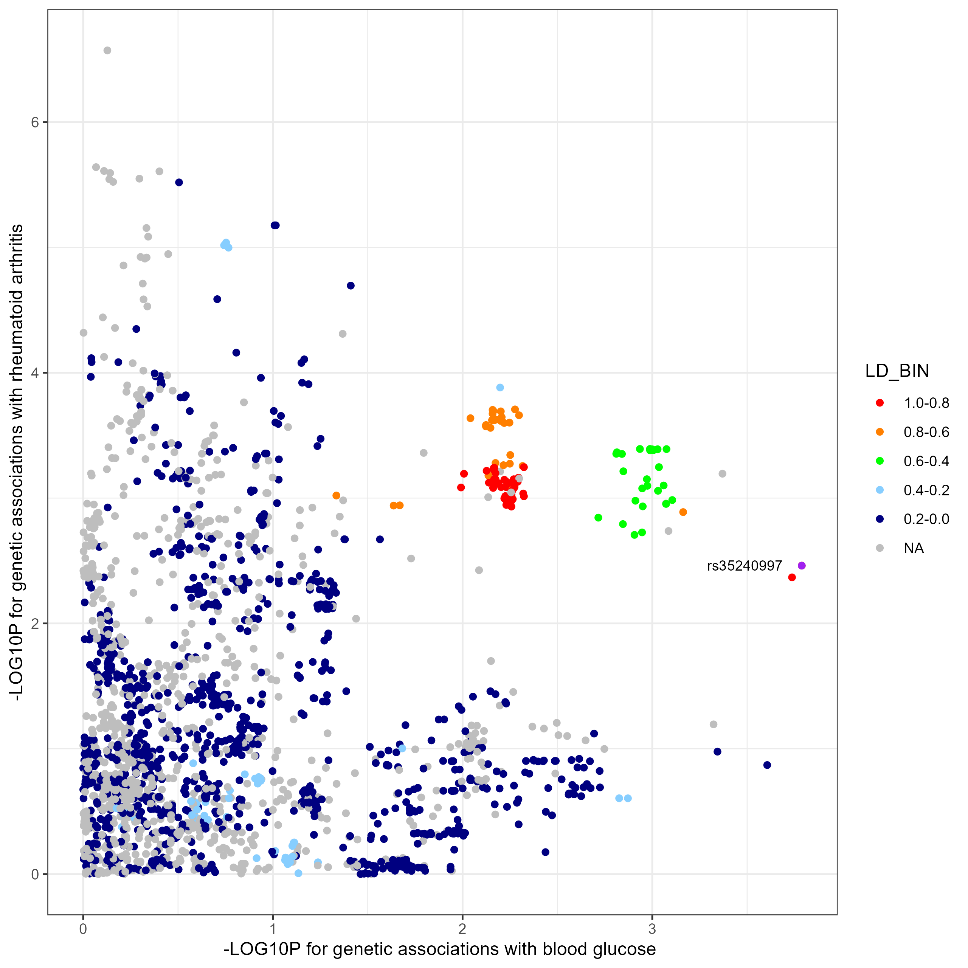


**Supplementary Fig. 4. Genetic associations with glucose concentration and rheumatoid arthritis within ±300kb of *PPARG* gene**

(A) Regional association plot, (B) Regional Q-Q plot

The purple dot represents the genetic instrument (rs35240997) of thiazolidinediones. Dots in other colors in the right panel of each plot represent the extent of linkage disequilibrium between the genetic instrument and other genetic variants.
